# Nondestructive Detection of Rice Milling Quality Using Hyperspectral Imaging with Machine and Deep Learning Regression

**DOI:** 10.3390/foods14111977

**Published:** 2025-06-03

**Authors:** Zhongjie Tang, Shanlin Ma, Hengnian Qi, Xincheng Zhang, Chu Zhang

**Affiliations:** 1 School of Information Engineering, Huzhou University, Huzhou 313000, China; 2 Institute of Crop Science, Huzhou Academy of Agricultural Sciences, Huzhou 313000, China

**Keywords:** hyperspectral imaging, multi-task learning, brown rice rate, milled rice rate, head rice rate

## Abstract

The brown rice rate (BRR), milled rice rate (MRR), and head rice rate (HRR) are important indicators of rice milling quality. The simultaneous detection of these three metrics holds significant economic value for rice milling quality assessments. In this study, hyperspectral imaging was employed to estimate the rice milling quality attributes of two rice varieties (Xiushui121 and Zhehujing26). Partial Least Squares Regression (PLSR), Support Vector Regression (SVR), Convolutional Neural Networks (CNNs), and Backpropagation Neural Networks (BPNNs) were used to establish both single-task and multi-task models for the prediction of milling quality attributes. Most multi-task models demonstrated a higher prediction accuracy compared with their corresponding single-task models. Among single-task models, BPNNs outperformed the others in predicting BRR and HRR, with correlation coefficients (*r*) up to 0.9. SVR excelled in forecasting the MRR. In multi-task learning, BPNNs exhibited relatively better performance, with *r* values exceeding 0.81 for all three indicators. SHapley Additive exPlanations (SHAP) analysis was used to explore the relationship between wavelength and rice milling quality attributes. This study confirmed that this nondestructive detection method for rice milling quality using hyperspectral imaging combined with machine learning and deep learning algorithms could effectively assess rice milling quality, thus contributing to breeding and growth management in the industry.

## 1. Introduction

Rice, as one of major food crops in the world, has a direct impact on consumer health and food security. Milling quality is a crucial indicator for rice milling performance evaluation, which is also a crucial indicator for rice quality evaluation. Rice milling quality primarily includes brown rice rate (BRR), milled rice rate (MRR), and head rice rate (HRR). During rice processing, the brown rice or paddy undergoes a series of steps, including hulling, milling, polishing, grading, and packaging, before being stored, distributed, and sold. Typically, when processing 100 kg of paddy, about 20% is separated as husk, leaving 80% as brown rice. Further milling of the brown rice results in approximately 10% bran and 70% milled rice [1]. The milled rice consists of a mixture of head rice and broken grains. High-quality milled rice requires that the proportion of broken grains in head rice does not exceed 4% [1]. Thus, only about 66% of the original paddy is suitable for consumption.

Head rice generally holds a higher economic value, while broken rice is commonly used in various processed products. Maintaining milling quality is essential for preserving the market value and usability of rice. Monitoring the degree of milling and minimizing the proportion of broken rice are crucial for reducing economic losses [2].

Traditional methods to assess rice milling quality, including manual sensory evaluation, chemical analysis, and physical indicator measurements, are often limited by a low efficiency, high operational complexity, and subjectivity. These approaches tend to be time-consuming, labor-intensive, and error-prone, making them inadequate for modern agricultural production and processing demands [3,4]. Conventional visual inspection methods exhibit limitations in distinguishing visually similar rice varieties, making it difficult to accurately identify quality differences, thereby affecting the quality control of the final product [5]. To overcome these shortcomings of traditional techniques, various spectral and imaging technologies, such as hyperspectral imaging (HSI), near-infrared spectroscopy, thermal imaging [6], and laser backscattering imaging, have been successfully applied to rice quality detection, significantly improving its accuracy and efficiency [7].

Hyperspectral imaging (HSI) has emerged as a rapid, nondestructive, and highly efficient detection tool, demonstrating substantial potential in agricultural product quality assessment [8]. This technology can simultaneously capture the spatial and spectral information of the tested object, enabling a comprehensive analysis of both internal composition and external characteristics. By analyzing the unique spectral signatures of materials, HSI has been widely validated for its effectiveness in agricultural and food quality detection [9]. A single scan enables the simultaneous acquisition of multi-dimensional data with a significantly higher efficiency than conventional chemical methods. Chen et al. proposed a hyperspectral imaging (HSI)-based method to quantify residual bran on milled rice grains, achieving a 93.5% accuracy in mapping bran distribution patterns [10].

Both traditional machine learning and deep learning techniques have achieved remarkable results in the field of rice quality detection using hyperspectral imaging. By leveraging the rich spectral information provided by hyperspectral imaging (HSI) technology, machine learning models can achieve the high-precision prediction of agricultural product quality attributes, demonstrating excellent performance in terms of accuracy and reliability for quality assessment. Yang et al. [9] employed an optimization approach combining cross-validation and grid search to construct an efficient CatBoost model, which achieved an accuracy of 91.31% in predicting the degree of milling (DOM) of rice. Meanwhile, as an advanced branch of machine learning, deep learning has exhibited outstanding performance in tasks such as image classification and object detection due to its capability for automatic feature extraction [11]. In particular, convolutional neural networks (CNNs) can directly learn multi-level feature representations from raw hyperspectral data without manual intervention, thereby effectively uncovering latent information within the data. Emerging deep learning technology brings new opportunities for effectively processing massive and diverse data from seeds and evaluating their quality [12]. Prabahar et al. [13] demonstrated that a CNN combined with near-infrared (NIR) spectroscopy can effectively screen rice quality. Their results showed that CNN-based regression models achieved more accurate quantitative estimations of amylose content and chalkiness compared to traditional regression approaches.

Current research on rice milling quality prediction primarily focuses on single-task detection, predicting an object one time (e.g., head rice rate), while an in-depth exploration of multi-task joint detection remains lacking [14]. Multi-task refers to the simultaneous detection of multiple objects. In reality, milling quality is a multi-dimensional composite metric involving multiple interrelated parameters, including brown rice rate, milled rice rate, and head rice rate. These parameters are not independent but may exhibit complex nonlinear relationships. Therefore, developing a multi-task joint detection method based on hyperspectral imaging and deep learning holds significant importance for achieving a comprehensive, accurate, and nondestructive assessment of rice milling quality. Such an approach not only improves detection efficiency and reduces computational costs [15] but also provides more holistic data support for quality control in rice processing.

This study aims to explore the feasibility of using hyperspectral imaging technology to simultaneously predict rice milling quality attributes (BRR, MRR, and HRR). By collecting hyperspectral images of different rice varieties under different treatments, the quantitative models between milling quality attributes and spectral features are established, providing a theoretical foundation and technical support for the rapid, nondestructive detection of rice milling quality. The specific objectives are (1) to develop single-task models—including Partial Least Squares Regression (PLSR), Support Vector Regression (SVR), Backpropagation Neural Networks (BPNNs), and Convolutional Neural Networks (CNNs), with each model predicting one quality indicator; (2) to construct multi-task models (PLSR, BPNN, and CNN) and a multi-output SVR for the simultaneous prediction of all three quality indicators, BRR, MRR, and HRR; (3) to employ SHapley Additive exPlanations (SHAP) for BPNNs’ visualization and interpretability analysis, enhancing understanding of the decision-making process of the models.

## 2. Materials and Methods

### 2.1. Materials and Experimental Design

Two japonica rice cultivars, Xiushui121 and Zhehujing26, were used in this study. Field experiments were conducted at the Balidian Experimental Station of the Huzhou Academy of Agricultural Sciences (30°48′36″ N, 120°11′24″ E) and Jianliang Family Farm (30°51′54″ N, 120°05′56″ E) in the Wuxing district of Huzhou, China, in 2023. The soil type at both sites was clay. The experimental design employed a split plot arrangement, with nitrogen fertilizer treatment as the main plot and variety as the subplot. There were three replicates for each treatment in Balidian Station and one replicate for each treatment in Jianliang Farm, respectively. A total of 16 treatments were established in two sites, with detailed information listed in Table S1 (in the Supplementary Materials). The topdressing fertilizers were applied at the tillering stage (I, 30 June 2023), panicle initiation stage (II, 9 August 2023), and booting stage (III, 18 August 2023), respectively.

At the rice maturity stage, approximately 100 continuous panicles in a row were collected in each plot. Then, the sample grains were dried naturally by sunlight. Then, the seeds of these panicles were threshed and mixed thoroughly. The seeds were then divided into five samples. In all, 240 samples (5 samples × 8 treatments × 2 varieties × 3 three replicates) and 80 samples (5 samples × 8 treatments × 2 varieties × 1 replicate) were obtained from Balidian Station and Jianliang Farm, respectively. Thus, a total of 320 samples were obtained in this study. However, hyperspectral images of 15 samples were missing due to machine issues, and only 305 samples were used for further analysis. In each sample, about 20 g of rice seeds was used for hyperspectral image acquisition and milling quality measurement.

### 2.2. Measurement of Rice Milling Quality Indicators

The BRR, MRR, and HRR are the key indicators of rice milling quality, and primary factors influencing its market price. These indicators were determined according to the methods of Dou et al. [16] with a minor modification. Approximately 20 g of paddy rice was dehulled to produce brown rice using a rice huller (JLG-IIA, Zhongchuliang, Chengdu, China). The milled rice was obtained from the brown rice using a milled rice machine (JNM-III, Zhongchuliang, Chengdu, China). The head rice was defined as that whose grain volume was over 3/4 of an intact grain body, and the assessment was conducted manually.

### 2.3. Hyperspectral Image Acquisition

The hyperspectral imaging system consisted of a camera, light source, motorized translation stage, and a computer. In this experiment, the hyperspectral camera (FX17, SPECIM, Oulu, Finland) acquired hyperspectral images in the near-infrared range (900–1700 nm). Rice seed samples were placed on the motorized stage at a distance of 30 cm from the camera. During the hyperspectral images’ collection, the stage moved at a constant speed of 26.3 mm·s^−1^. The hyperspectral images’ acquisition was controlled using the LUMO Scanner software 2020 (SPECIM, Oulu, Finland).

Spectral data from about 200 seeds were extracted from one hyperspectral image. For each sample, 5–7 hyperspectral images were acquired. To mitigate the effects of uneven illumination, lens transmission variations, and dark current noise from the camera, white and dark reference corrections were applied to the hyperspectral images prior to spectral extraction. The dark reference image (reflectance ≈ 0%) was acquired by covering the lens with its cap, while the white reference image (reflectance ≈ 100%) was obtained using a white Teflon board. The reflectance (R) was calculated using Equation (1):

(1) $$R=\frac{I-D}{W-D}$$

where *R* is the corrected reflectance data, *I* is the raw intensity data, *D* is the dark reference data, and *W* is the white reference data.

### 2.4. Spectral Extraction

During the acquisition of hyperspectral images, a corresponding RGB image with the same spatial size was also generated. Due to the fact that the seeds and the black background showed significant color differences, the RGB images were binarized to obtain a mask. In the mask, 8 connected neighborhoods were used to determine whether pixels belonged to the same region. The foreign materials (dust or others) in the masks were successfully removed by filtering all regions with a number of pixels less than 100. Finally, in the mask, each connected region represented a seed. Image masks were then applied to the hyperspectral images to separate the seed samples from the background. The spectral information of individual seeds was extracted according to the acquisition sequence, with the entire area of each seed defined as the region of interest (ROI). Each pixel within the area contained a spectrum. Finally, the average spectrum of each seed was derived by computing the average values of the spectra of all pixels within the ROI. There were five to seven hyperspectral images of each sample, and the spectra of all the seeds in one sample was averaged to one spectrum representing the sample. Thus, a total of 305 spectra were obtained for the 305 samples.

The raw spectral curves exhibited uninformative wavelengths at both ends, likely due to instrumental and environmental factors during measurement. To enhance the subsequent analysis and improve the modeling performance, preprocessing of the raw spectral data was necessary. In this study, the noisy bands at both ends of the spectrum were selectively removed before the spectral extraction based on the preview of the seed spectral profiles. The first 13 spectral bands at the beginning of the spectra (below 980 nm) and the last 11 bands at end of the original spectra (beyond 1684 nm) were excluded, resulting in an effective spectral range of 980–1684 nm for further analysis. The remaining spectra contained 200 bands.

### 2.5. Regression Analysis Methods

To build the regression models, the independent variable X consisted of spectral data, and the dependent variables Y were the three quality indicators: BRR, HRR, and MRR. The regression models were built without special labels.

#### 2.5.1. Partial Least Squares Regression

Partial Least Squares Regression (PLSR) is a multivariate statistical data analysis method capable of performing regression modeling (multivariate linear regression), data structure simplification (principal component analysis), and correlation analysis between variable sets (canonical correlation analysis). By projecting high-dimensional data spaces of both independent and dependent variables into corresponding low-dimensional spaces, it derives mutually orthogonal feature vectors for each, then establishes univariate linear regression relationships between these feature vectors [6]. This approach not only addresses collinearity issues but also emphasizes the explanatory and predictive power of independent variables on dependent variables during feature selection, effectively eliminating noise irrelevant to the regression and resulting in models with minimal variables. Optimal parameters were selected using grid search and 10-fold cross-validation, with principal components optimized within the range of 1–20.

#### 2.5.2. Support Vector Machine

Support Vector Machine (SVM) is a classical machine learning algorithm widely applied to classification and regression tasks. Its core concept involves constructing an optimal hyperplane for data classification or regression. SVM demonstrates excellent performance in handling high-dimensional data and nonlinear problems, particularly maintaining a strong generalization capability even with small sample sizes [17]. Support Vector Regression (SVR), an extension of SVM for regression problems, aims to predict continuous values through the construction of regression functions. As a powerful regression tool, SVR has shown unique advantages in hyperspectral data analysis. It effectively processes high-dimensional, nonlinear spectral data and adapts flexibly to different data distributions through kernel functions. For parameter optimization, grid search and 10-fold cross-validation were employed on the validation set, with kernel function types including linear, radial basis function (rbf), and polynomial (poly). The regularization parameter ranged from 10^−7^ to 10^7^ across 14 orders of magnitude, while kernel coefficients used both ‘scale’ and ‘auto’ modes.

#### 2.5.3. Backpropagation Neural Network

The Backpropagation Neural Network (BPNN) is a classic multilayer feedforward neural network widely used for pattern recognition and regression prediction tasks. The BPNN adjusts network weights and biases through the backpropagation algorithm, employing mean squared error (MSE) as the loss function to optimize model performance [18]. In this study combining hyperspectral imaging with deep learning for the nondestructive detection of rice milling quality, the BPNN utilizes feature vectors extracted from hyperspectral data as input. Through nonlinear transformations across multiple hidden layers, it extracts spectral features and predicts key quality indicators (brown rice rate, milled rice rate, and head rice rate) at the output layer.

The BPNN architecture consists of two fully connected layers, including an input layer, hidden layer, and output layer. The input layer size was set to 200, with hidden layer dimensions of 1624 for brown rice rate prediction and 1024 for both milled rice rate and head rice rate predictions. For brown rice rate modeling, the training parameters included 6000 epochs, a batch size of 16, and a learning rate of 0.001. The milled rice rate and head rice rate models were trained for 5000 epochs, with a batch size of 32 and learning rate of 0.001. The Rectified Linear Unit (ReLU) activation function was employed in the hidden layers, and the Adam optimizer was utilized to enhance the regression’s prediction accuracy, thereby optimizing the prediction performance for rice milling quality.

During the training of BPNN models, the models and the corresponding results of each epoch were saved. After the training, the models with optimal performance were selected for further analysis.

#### 2.5.4. Convolutional Neural Network

In the nondestructive detection of rice milling quality using hyperspectral imaging combined with deep learning, Convolutional Neural Networks (CNNs) can be applied not only for classification tasks but also for regression tasks to predict continuous variables of milling quality (brown rice rate, milled rice rate, and head rice rate). CNNs establish precise regression predictions by extracting features from hyperspectral data and learning the mapping relationships between these features and quality indicators [19]. The CNN architecture primarily consists of the following key components: an input layer, convolutional layers, pooling layers, fully connected layers, and output layer. Compared with traditional spectral regression methods, CNNs demonstrate superior nonlinear modeling capabilities, enabling the automatic learning of complex features from high-dimensional spectral data to improve the accuracy and robustness of rice milling quality predictions.

CNNs can effectively implement multi-task learning by jointly optimizing correlations between multiple tasks, thereby enhancing model training efficiency. We designed a multi-output model where each output corresponds to a specific task, allowing the model to share underlying convolutional layers and feature extractors while generating separate prediction results for each task.

**Figure 1:**
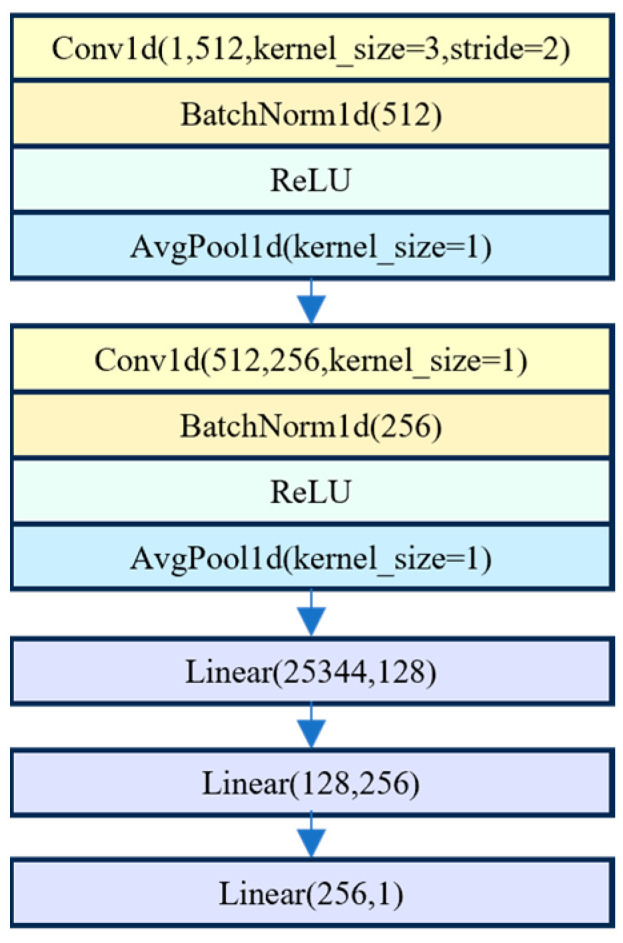
Architecture of the 1D-CNN model.

In this study, a one-dimensional CNN (1D-CNN) was used. For spectral data analysis using 1D-CNN, local features such as absorption peaks and changes in reflectance within a specific wavelength range could be identified by sliding along the spectra with a one-dimensional convolution kernel. The activation function and multi-layer stacked structure were used to construct a complex nonlinear mapping. Thus, 1D-CNN was used to extract localized spectral features and manage nonlinear spectral dependencies, even in the absence of spatial structure. The CNN model was constructed using the PyTorch 1.11.0 framework, with ReLU activation functions employed in the convolutional layers. The CNN model for predicting brown rice rate, milled rice rate, and head rice rate comprises five layers, including two convolutional layers and three fully connected layers. The overall architecture of the CNN model is illustrated in Figure 1. The batch size was set to 8 for the brown rice rate prediction and 16 for both milled rice rate and head rice rate predictions. The learning rate (LR) was established at 0.001, with the Adam optimization algorithm employed to minimize loss during model training.

During the training of the CNN models, the models and the corresponding results of each epoch were saved. After the training, the models with optimal performance were selected for further analysis.

#### 2.5.5. Multi-Task Learning

Multi-task learning (MTL) is a machine learning method that simultaneously optimizes multiple related tasks by sharing feature representations, thereby enhancing models’ generalization and prediction accuracy. In this study of nondestructive rice milling quality detection combining hyperspectral imaging and deep learning, an MTL framework was adopted to concurrently predict brown rice rate, milled rice rate, and head rice rate. This approach fully utilizes the multidimensional information in hyperspectral data to improve modeling performance. Four MTL methods were implemented:

Multi-Task PLSR: This method extracts latent factors from hyperspectral data through linear regression modeling while simultaneously predicting multiple target variables [20]. During training, the number of principal components were optimized within the range of 1–20.

Multi-Output Support Vector Machine (Multi-Output SVM or Multi-Target SVM) extends the traditional SVM to address multi-output (multi-target) regression or classification problems [21]. This approach is particularly suitable for scenarios requiring the simultaneous prediction of multiple correlated indicators with limited sample sizes.

Multi-Task BPNN: The BPNN utilizes a multilayer feedforward structure, optimizing weights for multiple target variables through error backpropagation, while learning correlations between different quality indicators to improve its nonlinear fitting performance [22].

Multi-Task CNN: The CNN architecture employs convolutional layers to extract both local and global features from hyperspectral data, achieving the joint learning of multiple quality indicators through shared network structures. This approach effectively explores deep spectral features to enhance its prediction capability [23].

Compared with single-task learning, MTL establishes connections among related quality indicators, reduces overfitting through information sharing, enhances model generalization, and delivers more stable and reliable predictions for rice milling quality. As for the multi-task learning models, mean squared error (MSE) was used as the loss function. The weights of the loss of the three tasks (the prediction of BRR, MRR, and HRR) were kept equal during the training, and the weights were not tuned.

### 2.6. SHAP Analysis

SHAP (SHapley Additive exPlanations) analysis, grounded in game theory, was employed to interpret the optimal models by quantifying the contribution of each input feature (SHAP value) based on its average impact on model outputs [24]. In this study, SHAP values were calculated using all the samples from the training sets of both single-task BPNN and multi-task BPNN models, as the backpropagation-based regression networks demonstrated superior performance and were selected for SHAP interpretation. Summary plots were generated for the single-task and multi-task training sets to visualize the magnitude and direction of influential spectral bands.

### 2.7. Dataset Construction and Outlier Sample Removal

As mentioned above, the average spectrum of each sample was obtained, and the BRR, MRR and HRR values of the corresponding sample were measured. The spectral data and the milling quality indicators were then used to establish calibrations. To ensure a robust model performance for predicting brown rice rate, milled rice rate, and head rice rate, outliers and anomalous samples that could compromise model accuracy were removed. During the measurement of milling quality indicators, three samples were mismeasured. The remaining 302 samples were then used for further analysis. A boxplot method was used to identify samples with extreme values of the three indicators (in Figure S1), and two samples with extreme values were identified as outlier samples. Then, PLSR models were established using all the samples for the three milling quality indicators, and the samples with a larger prediction error were manually identified as outlier samples. In all, 18 additional outliers were identified for the three milling quality indicators. It should be noted that for one milling quality indicator, the samples identified as outlier samples were also removed for the other two indicators. Thus, after removing 23 anomalous samples, the remaining 282 samples were retained for modeling. Then, the samples were randomly split into the training, validation, and test sets at the ratio of 4:1:1, and no samples repeatedly occurred in the three sets. The dataset split was not stratified by treatment or variety. The samples in the three sets for the three milling quality indicators were the same. Moreover, the samples in the three sets for single-task and multi-task learning were the same. Thus, the model performance could be better compared. The statistical analysis of the milling quality indicators in the training, validation, and test sets is shown in Table 1.

### 2.8. Model Evaluation

To systematically evaluate the predictive performance of the four models—Partial Least Squares Regression (PLSR), Support Vector Regression (SVR), Convolutional Neural Network (CNN), and Backpropagation Neural Network (BPNN)—on both single-task and multi-task scenarios, this study employed two key evaluation metrics: (1) the correlation coefficient (*r*) between predicted and true values, which measures the linear goodness-of-fit of the model; and (2) the root mean square error (RMSE) between predicted and true values, which assesses the predictive accuracy of the model. For each model, these two metrics were calculated on the training set, validation set, and test set. A value of *r* closer to 1 indicated better model fitting, while an RMSE closer to 0 signified a smaller prediction error. A model with a higher *r* and lower RMSE was treated as a better model.

### 2.9. Software and Hardware

All data processing and model development were performed on computer systems equipped with 32 GB RAM, an NVIDIA GeForce RTX 3060 GPU, and Intel i7-11700 CPU. The computational environment utilized Python (v3.9), with PyCharm IDEs (1 March 2022) as the programming platforms.

The data extraction and SHAP analysis were implemented in Python, while the machine learning algorithms were performed using scikit-learn and PyTorch 1.11.0. For deep learning, the PyTorch framework was employed.

## 3. Results

### 3.1. Spectral Analysis

**Figure 2:**
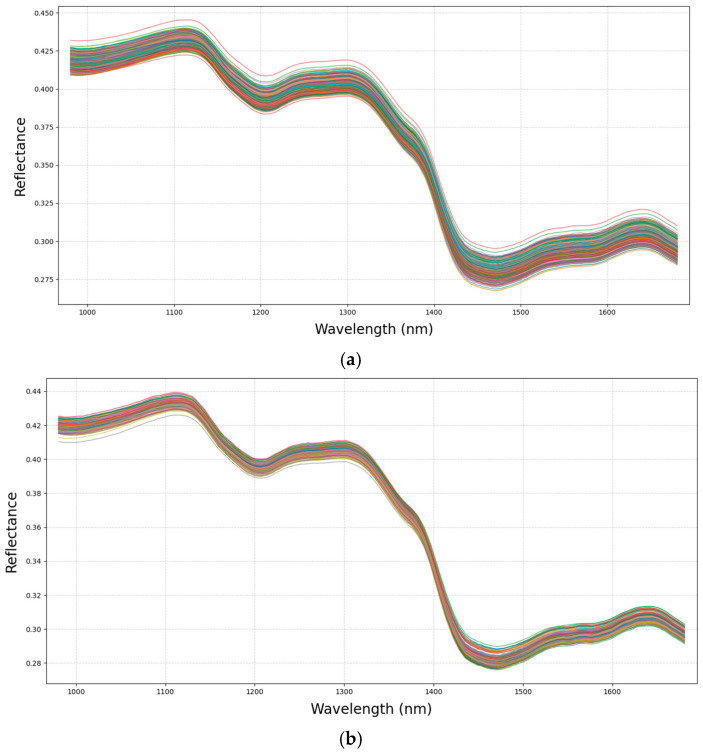

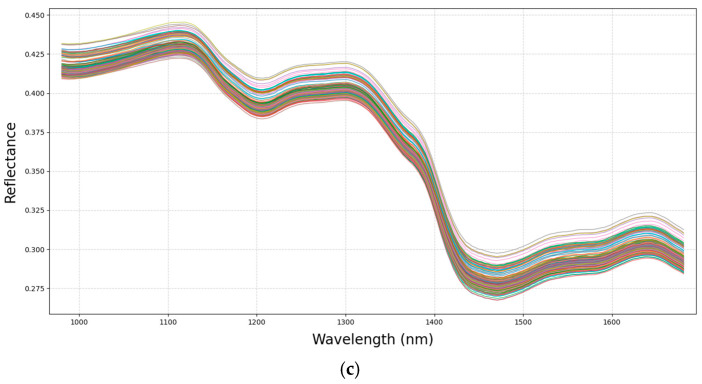
(**a**) Average reflectance spectra of rice seeds. (**b**) Average reflectance spectra of Xiushui121. (**c**) Average reflectance spectra of Zhehujing26.

The milling quality of rice is primarily determined by three parameters, brown rice rate, milled rice rate, and head rice rate, which can be reflected in spectral reflectance characteristics. Figure 2 displays the average reflectance spectra of rice seed samples from two varieties across the 980–1684 nm spectral range. The x-axis represents the wavelength range, while the y-axis indicates the corresponding spectral reflectance. As shown in Figure 2, the spectral curves of the two rice varieties exhibited minimal divergence within the same spectral range due to their similar biochemical compositions. The overall trends of the reflectance spectra were consistent, with matching peak and trough positions across both varieties. Notably, a distinct reflectance peak was observed near 1120 nm for all rice seed samples.

### 3.2. Performance of Single-Task and Multi-Task Models

#### 3.2.1. Single-Task Model Results

In this study, the traditional machine learning (PLSR, SVR, BPNN) and deep learning (CNN) methods were used to establish a single-task model to predict the brown rice rate, milled rice rate, and head rice rate of rice seeds. The prediction results of single-task PLSR, SVR, BPNN and CNN models for brown rice rate, milled rice rate, and head rice rate are shown in Table 2.

The established single-task PLSR models were compared and analyzed. The correlation coefficients of the three prediction sets for brown rice rate, milled rice rate, and head rice rate were 0.828, 0.725, and 0.730, respectively. The performance for brown rice rate was the best. The established single-task SVR models were compared and analyzed. The correlation coefficients of the three prediction sets for brown rice rate, milled rice rate, and head rice rate were 0.856, 0.779, and 0.833, respectively. SVR models for brown rice rate and head rice rate performed best. In the single-task CNN model, the correlation coefficients of the three prediction sets for brown rice rate, milled rice rate, and head rice rate were 0.846, 0.772, and 0.756, respectively. CNN model for brown rice rate performed slightly better than the models for the other two indicators. The results of the CNN model were slightly better than PLSR. In the single-task BPNN model, the correlation coefficients of the three prediction sets for brown rice rate, milled rice rate, and head rice rate were 0.865, 0.768 and 0.840, respectively. The results of the BPNN model were similar to those of SVR. The BPNN performed better than SVR in brown rice rate and head rice rate, and the BPNN performed close to SVR in milled rice rate. For BRR, PLSR performed relatively worse compared to other models, while the BPNN yielded better results. For MRR, SVR showed the best performance, whereas PLSR performed relatively worse. For HRR, the BPNN achieved the best results, and PLSR performed relatively worse than the other models.

On the whole, the results of the CNN were better than those of PLSR, and the results of SVR and the BPNN were close to and higher than those of the CNN. The prediction of brown rice rate achieved good results by PLSR, SVR, the CNN, and the BPNN. The prediction results of milled rice rate by SVR, the CNN and the BPNN were similar, and the results of PLSR were poor. The results of PLSR and the CNN were lower than those of SVR and the BPNN.

As shown in Table 2, the training time of different models varied. The training time of the PLSR model was the shortest. The training time of PLSR models for the three milling quality indicators were close, and the training time of SVR models for the three milling quality indicators were also close. The close training time was attributed to the similar amount of data, model parameter settings, and optimization procedure. The training time of CNN models as well as BPNN models for different milling quality attributes were different, and the differences were determined by the model structure and parameters. The variations in training time implied the influence of different model training procedures, different model parameters settings, and different model-tuning procedures.

**Figure 3:**
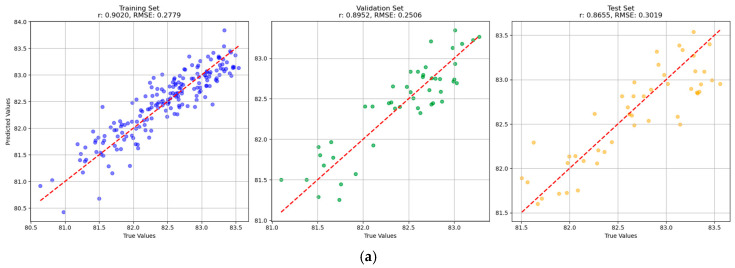

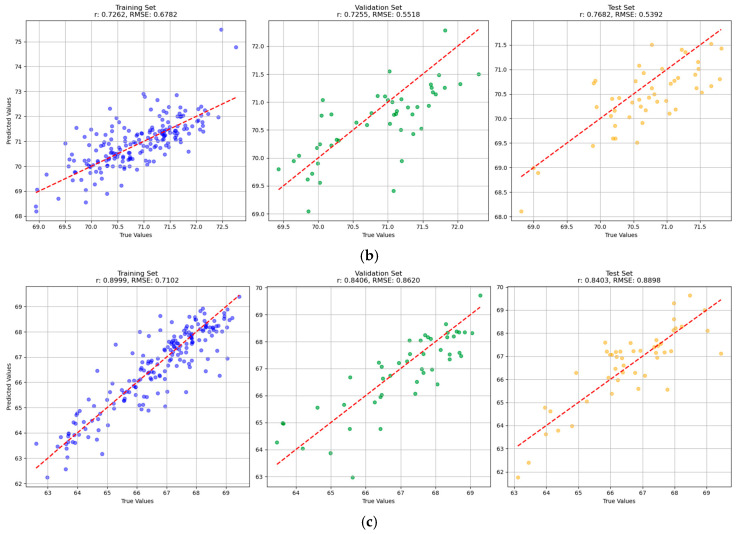
Scatter plots of predicted versus actual values for (**a**) brown rice rate, (**b**) milled rice rate, and (**c**) head rice rate using the single-task BPNN model. The units in the horizontal axis and the ordinate axis are %.

The overall prediction performance of single-task BPNN models was relatively better than the other models, and the results of the BPNN are intuitively displayed to show the fitting effect of the optimal model by drawing a scatter diagram of the predicted and true values of the training set, validation set, and test set (Figure 3). The measured (true) values and predicted values of BRR, MRR, and HRR for samples in the test set of single-task BPNN models are presented in Figure S2. It can be found that the general differences between the measured values and the corresponding predicted values were not large, illustrating the prediction performance of the models.

#### 3.2.2. Results of the Multi-Task Model

The prediction results of the multi-task models are shown in Table 3. The prediction accuracy of the multi-task PLSR model in the three prediction sets of brown rice rate, milled rice rate, and head rice rate was 0.816, 0.728, and 0.730, respectively, and the prediction performance of brown rice rate was the best (Table 4). The prediction accuracy of the multi-task CNN model in the three prediction sets was 0.859, 0.750, and 0.748, respectively, and the prediction performance of brown rice rate was still the best. The prediction accuracy of the multi-task BPNN model in the three prediction sets was 0.811, 0.819, and 0.870, respectively. The brown rice rate, milled rice rate, and head rice rate all achieved good prediction results. The prediction accuracy of the multi-output SVR model in the three prediction sets was 0.865, 0.774, and 0.841, respectively. For BRR, the multi-output SVR delivered better results, while PLSR and the BPNN performed relatively worse. For MRR, the BPNN achieved the best performance, whereas PLSR performed worse than the other models. For HRR, the BPNN yielded the best results, while PLSR and the CNN showed relatively poorer performance compared to the other models.

Among the three multi-task models and the multi output SVR model, the multi-task BPNN achieved the relatively better prediction performance. The prediction results of the three milling quality indicators were good and close. The overall results of the multi-task BPNN were slightly better than those of the four single-task models. The results of the multi-task CNN were slightly better than those of the multi-task PLSR, and the results of the multi-output SVR showed an *r_p_* of 0.865 in brown rice rate. On the whole, the performance of the multi-task BPNN obtained the overall better performance in the three indicators (brown rice rate, milled rice rate, and head rice rate).

As shown in Table 3, the training time of different models varied. The training time of the PLSR model was the shortest, and the BPNN model had the longest training time. However, the training time of SVR was longer than the CNN model. The different ways to tune the models and the different parameters were the main reason for the varied training times of different models. Overall, the training times of the multi-task models were longer than the corresponding single-task models, indicating the greater complexity of the training of the multi-task models. It should be noted that for both single-task learning models and multi-task learning models, although the training time varied, once the models were established, the prediction was quite fast (less than 1 s). Although the prediction times were different, they were all fast in terms of prediction, and the training time variations might not affect the real-world applications of these models.

**Figure 4:**
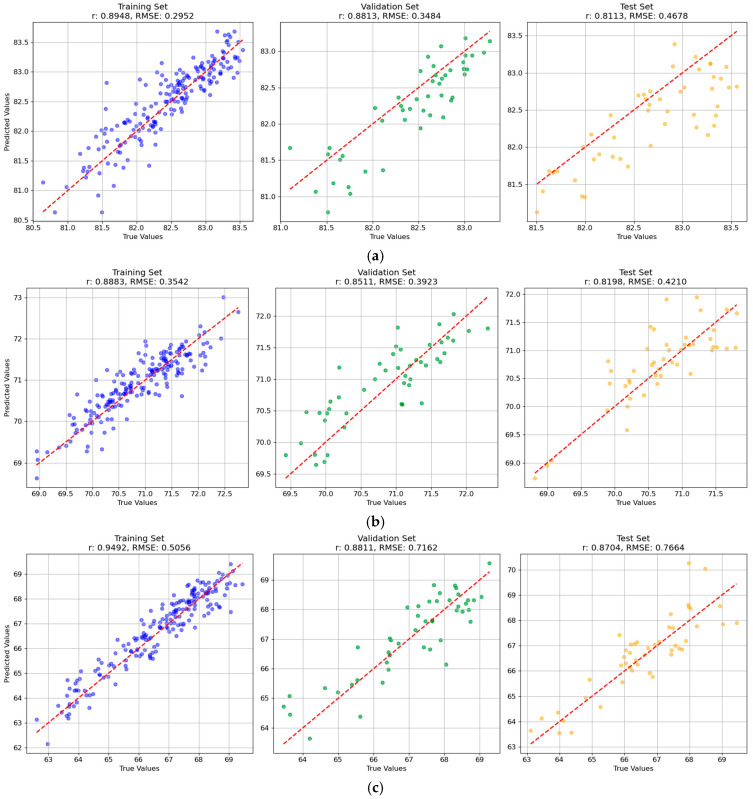
Scatter plots of predicted versus actual values for (**a**) brown rice rate, (**b**) milled rice rate, and (**c**) head rice rate using the multi-task BPNN model. The units in the horizontal axis and the ordinate axis are %.

The overall prediction performance of the multi-task BPNN models were relatively better than the other models, and the results of the BPNN are intuitively displayed to show the fitting effect of the optimal model by drawing a scatter diagram of the predicted and true values of the training set, validation set, and test set (Figure 4). The measured (true) values and predicted values of BRR, MRR, and HRR for samples in the test set of the multi-task BPNN model are presented in Figure S3. It can be found that the general differences between the measured values and the corresponding predicted values were not large, illustrating the prediction performance of the models.

### 3.3. Wavelength Importance Analysis

**Figure 5:**
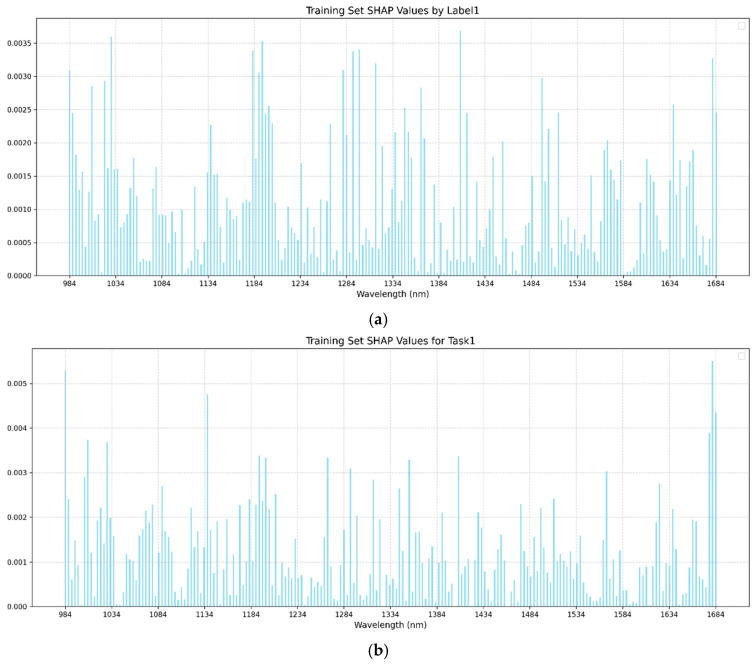
(**a**) SHAP chart of brown rice rate of single-task BPNN. (**b**) SHAP chart of brown rice rate of multi-task BPNN. The abscissa in the figure is the wavelength, and the ordinate is the SHAP value of the corresponding wavelength.

**Figure 6:**
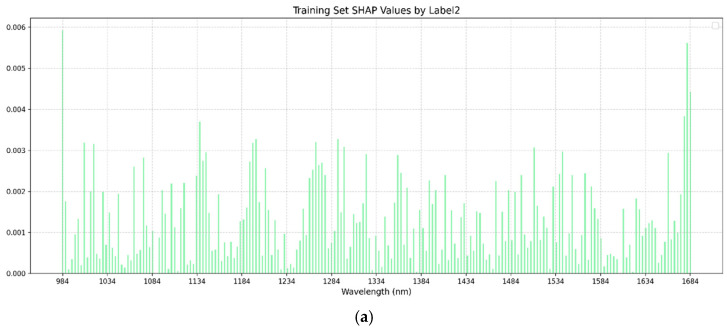

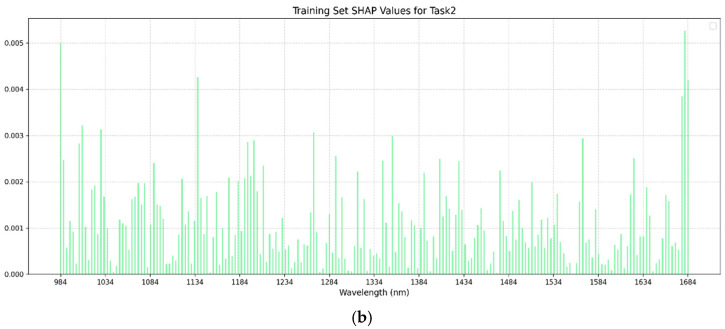
(**a**) SHAP chart of milled rice rate of single-task BPNN. (**b**) SHAP chart of milled rice rate of multi-task BPNN. The abscissa in the figure is the wavelength, and the ordinate is the SHAP value of the corresponding wavelength.

**Figure 7:**
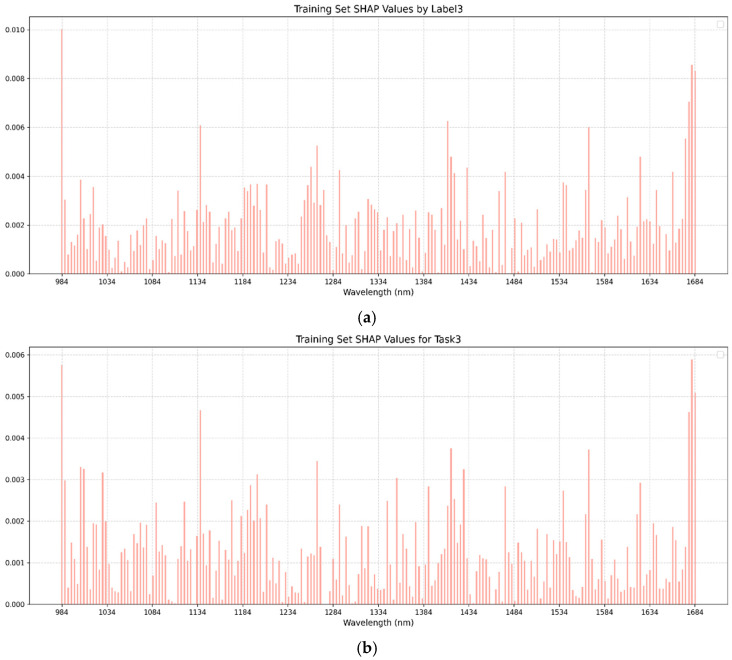
(**a**) SHAP chart of head rice rate of single-task BPNN. (**b**) SHAP chart of head rice rate of multi-task BPNN. The abscissa in the figure is the wavelength, and the ordinate is the SHAP value of the corresponding wavelength.

In this study, the SHAP analysis was used to visualize the single-task and multi-task BPNN models. In both single-task and multi-task learning models, the training sets of brown rice rate, milled rice rate, and head rice rate were selected for the SHAP method to visualize the analysis of feature importance. Figure 5, Figure 6 and Figure 7 present the SHAP value distributions for brown rice rate, milled rice rate, and head rice rate using single-task and multi-task learning. The abscissa is the wavelength, and the ordinate is the SHAP value of the corresponding wavelength. Table 4 displays the top 20 bands with the highest mean absolute SHAP values corresponding to BRR, MRR, and HRR in the single-task and multi-task learning models. By comparing the SHAP visualization results between single-task and multi-task learning, the differences in feature contributions to the three key rice milling quality indicators under different modeling strategies could be clearly observed.

According to the results presented in Table 4 and Figure 5, the feature wavelengths corresponding to the top 20 mean absolute SHAP values for BRR, MRR, and HRR exhibited consistency between single-task and multi-task models, particularly in the top 10 key bands. For clarity, identical bands in Table 4 are shown in bold, while similar (but non-identical) bands were not marked. Under the multi-task learning framework, characteristic bands such as 1681 nm, 984 nm, 1137 nm, 1677 nm, and 1200 nm frequently appeared across different milling quality indicators (BRR, MRR, and HRR), indicating a distinct feature-sharing tendency in the multi-task model. In contrast, the overlap of top 20 feature bands in single-task models across different metrics was relatively low, suggesting a stronger inclination toward task-specific feature extraction. This feature substitution phenomenon implies that multi-task learning might achieve a more comprehensive feature representation.

To validate the reliability of the SHAP feature importance analysis, a Jaccard similarity analysis was additionally performed. The results (shown in Figure S4) demonstrated that among different tasks, the HRR model displayed the highest similarity in its top 20 important features (SHAP values, with the maximum Jaccard index), followed by the MRR model, while the BRR model showed the lowest feature similarity.

Further comparative analysis revealed that the multi-task model, by sharing critical feature bands, could more effectively capture the common patterns of rice quality parameters. In contrast, the single-task model, due to its excessive focus on modeling individual metrics, might overlook the global correlations among different quality parameters during feature selection. This finding might provide empirical evidence supporting the advantages of multi-task learning in spectroscopic analysis.

The SHAP analysis revealed that the importances of spectral bands were different, and some bands were more important, which may be related to the spectral response characteristics of the milling quality indicators in the data, helping to reveal the theoretical explanation for using spectral characteristics to predict milling quality indicators. These results provided a basis for the further optimization of the feature selection or interpretation of the models.

## 4. Discussion

This study used hyperspectral imaging combined with machine learning and deep learning for the nondestructive evaluation of rice milling quality (brown rice rate, milled rice rate, and head rice rate). Overcoming the limitations of traditional destructive methods, our approach enabled the efficient quality assessment crucial for optimizing milling processes, enhancing commercial value, and meeting precision agriculture requirements.

Hyperspectral imaging has emerged as a powerful tool for agricultural products’ quality assessment due to its ability to capture detailed spectral signatures correlated with physical characteristics. In this study, machine learning and deep learning approaches were employed to predict rice milling quality with good performances. Compared with existing studies, our results exhibited certain differences and advantages. Specifically, Ageh et al. [1] achieved prediction accuracy using a random forest model, with R² values of 0.801 for BRR, 0.714 for MRR, and 0.995 for HRR. Ebrahim et al. [25] used an artificial neural network to predict the HRR, with the R^2^ value of as high an accuracy as 0.98. In contrast, our study obtained correlation coefficients across training, validation, and test sets as follows: BRR (0.894, 0.881, 0.811), MRR (0.888, 0.851, 0.819), and HRR (0.949, 0.881, 0.870). Comparative analysis indicated that our BRR and HRR predictions were slightly lower than those of Ageh et al. [1], while the MRR results were comparable. These discrepancies may be attributed to differences in sample processing methods, experimental design, and feature distribution. Furthermore, when compared with the near-infrared spectroscopy-based approach by Sun et al. [26] (BRR: calibration R^2^ = 0.68, validation R^2^ = 0.65; MRR: calibration R^2^ = 0.71, validation R^2^ = 0.70; HRR: calibration R^2^ = 0.69, validation R^2^ = 0.67), our BRR results were similar, the MRR was marginally lower, and the HRR demonstrated superior performance. These findings suggested that the methodology adopted in this study was effective for assessing BRR, MRR, and HRR as key milling quality indicators. At present, there are few relevant studies, and more extensive studies are needed.

As presented in this study, the machine learning and deep learning models obtained differentiated performances. By comparing all the models, BPNN models obtained relatively better performances, and CNN models did not obtain the best performances. In some other studies, a similar phenomenon could be found that deep learning models did not exhibit better performances [27,28]. Multiple factors could potentially influence the prediction accuracy, including data distribution patterns, feature cluster selection, and model parameter configuration [9]. The predictive performance of CNN models in this study exhibited limitations, primarily attributable to the following: (1) a data–model architecture mismatch phenomenon, where the spatial distribution characteristics of spectral features demonstrated incompatibility with the CNN local receptive field mechanism [29]; and (2) a model complexity–data scale disparity, as CNN training required substantial datasets, whereas the currently available sample size remains constrained under existing experimental conditions [27].

In this study, the single-task learning (STL) and multi-task learning (MTL) prediction models were constructed using machine learning and deep learning algorithms. The results showed that although the two modeling methods were similar in prediction accuracy, the multi-task learning model showed a better overall performance. The multi-task BPNN model obtained relatively better overall performances among all the models. The results of single-task and multi-task were different with different indicators and different models. As mentioned above, MTL was not universally superior to single-task learning (STL). Differentiated results for STL and MTL could also be found in the literature [23,30,31,32]. In these studies, the results of STL and MTL were also quite close. The advantage of MTL was obvious that it could achieve multi-tasks simultaneously, simplifying the prediction procedures and being more applicable for real-world applications. Although MTL could enhance data efficiency by leveraging inter-task correlations, task interference might degrade its performance. Thus, it is quite important to design the optimal learning strategies and optimal objective functions for MTL. The utilization of STL and MTL could be considered based on practical scenarios, regarding the generally close performances of STL and MTL.

Future research could be developed in the following directions: At the data level, the generalization ability of the model can be enhanced by expanding the dataset scale and improving data diversity; in terms of model architecture, more efficient network structure designs and parameter optimization methods can be explored; for multi-task learning, the focus can be on studying combination strategies for different loss functions and dynamic weight allocation mechanisms to further improve model performance. Further investigations should be conducted to improve the model performance and generalization ability for real-world applications.

## 5. Conclusions

This study established various models, including single-task PLSR, multi-task PLSR, single-task SVR, multi-output SVR, single-task CNN, multi-task CNN, single-task BPNN, and multi-task BPNN, to estimate the brown rice rate, milled rice rate, and head rice rate of different rice varieties. Overall, for BRR, multi-output SVR performed better in multi-task scenarios, while the multi-task PLSR and multi-task BPNN showed poorer performance. Single-task PLSR was generally worse compared to the other models, whereas the single-task BPNN performed better and was comparable to multi-output SVR. For MRR, SVR achieved the best performance in single-task scenarios, while single-task PLSR obtained a relatively poor performance. In multi-task scenarios, the BPNN performed the best, and the performance of PLSR was generally worse compared to the other models. The results for MRR in multi-task scenarios were better than those in single-task scenarios. For HRR, the BPNN yielded the best results in single-task scenarios, while PLSR performed relatively poorly compared to the other models. In multi-task scenarios, the BPNN performed the best, while PLSR and the CNN showed a relatively poorer performance compared to the other models. The results for HRR in multi-task scenarios were relatively better than those in single-task scenarios. Among the three milling quality indicators, milled rice rate predictions were relatively lower but could be improved in future studies through feature selection techniques and model optimization. The integration of hyperspectral imaging and deep learning showed tremendous potential for nondestructive rice milling quality detection.

## Figures and Tables

**Table 1:** Statistical characteristics of BRR, MRR, and HRR yield in training, validation, and test sets.

	Label	Max	Min	Average
Training	BRR	83.540	80.638	82.460
	MRR	72.754	68.943	70.867
	HRR	69.445	62.606	66.675
Validation	BRR	83.275	81.1	82.410
	MRR	72.3	69.430	70.845
	HRR	69.276	63.454	67.052
Test	BRR	83.577	81.505	82.651
	MRR	71.814	68.815	70.672
	HRR	69.445	63.110	66.577

**Table 2:** Prediction performance of single-task models for rice seed quality parameters.

Model	Training Time	Label	Training		Validation		Test	
			*r_c_* ^a^	RMSEC	*r_v_*	RMSEV	*r_p_*	RMSEP
PLSR	1.312 s	BRR	0.892	0.286	0.862	0.291	0.828	0.335
	1.314 s	MRR	0.758	0.493	0.758	0.477	0.725	0.516
	1.281 s	HRR	0.839	0.857	0.825	0.868	0.730	1.024
SVR	181.680 s	BRR	0.906	0.271	0.871	0.278	0.856	0.309
	196.622 s	MRR	0.790	0.465	0.763	0.466	0.779	0.450
	194.390 s	HRR	0.872	0.775	0.841	0.820	0.833	0.810
CNN	397.027 s	BRR	0.909	0.402	0.875	0.364	0.846	0.478
	118.705 s	MRR	0.797	0.463	0.745	0.488	0.772	0.456
	216.034 s	HRR	0.835	1.316	0.808	1.483	0.756	1.288
BPNN	334.098 s	BRR	0.902	0.277	0.895	0.250	0.865	0.301
	102.112 s	MRR	0.726	0.678	0.725	0.551	0.768	0.539
	96.370 s	HRR	0.899	0.710	0.840	0.862	0.840	0.889

a: *r_c_*, *r_v_*, and *r_p_* are the correlation coefficients for the training set, validation set, and test set, respectively. RMSEC, RMSEV, and RMSEP are the RMSE values for the training set, validation set, and test set, respectively.

**Table 3:** Comparative performance of multi-task models and multi-output SVR for rice quality prediction.

Model	Training Time	Label	Training		Validation		Test	
			*r_c_* ^a^	RMSEC	*r_v_*	RMSEV	*r_p_*	RMSEP
PLSR		BRR	0.895	0.282	0.856	0.296	0.816	0.348
	3.143 s	MRR	0.770	0.482	0.787	0.449	0.728	0.511
		HRR	0.830	0.878	0.819	0.882	0.730	1.017
SVR		BRR	0.943	0.211	0.873	0.297	0.865	0.281
	595.692 s	MRR	0.875	0.369	0.819	0.399	0.774	0.463
		HRR	0.872	0.775	0.833	0.810	0.841	0.820
CNN		BRR	0.857	0.546	0.862	0.587	0.859	0.466
	243.186 s	MRR	0.790	1.042	0.776	1.113	0.750	1.197
		HRR	0.831	0.912	0.791	0.941	0.748	0.989
BPNN		BRR	0.894	0.295	0.881	0.348	0.811	0.467
	1953.404 s	MRR	0.888	0.354	0.851	0.392	0.819	0.421
		HRR	0.949	0.505	0.881	0.716	0.870	0.766

a: *r_c_*, *r_v_*, and *r_p_* are the correlation coefficients for the training set, validation set, and test set, respectively. RMSEC, RMSEV, and RMSEP are the RMSE values for the training set, validation set, and test set, respectively.

**Table 4:** Wavelengths corresponding to the top 20 SHAP values for brown rice rate, milled rice rate, and head rice rate in single-task and multi-task learning.

BRR		MRR		HRR	
Single-Task	Multi-Task	Single-Task	Multi-Task	Single-Task	Multi-Task
**1407**	**1681**	**984**	**1681**	**984**	**1681**
**1029**	**984**	**1681**	**984**	**1681**	**984**
**1193**	1137	**1684**	**1137**	**1684**	**1684**
1298	**1684**	**1677**	**1684**	**1677**	**1137**
1182	1677	**1137**	**1677**	1411	**1677**
**1291**	**1008**	**1291**	**1008**	**1137**	**1414**
**1681**	**1029**	**1200**	1029	**1567**	**1567**
**1316**	**1193**	**1266**	**1266**	1674	**1266**
1280	**1407**	**1008**	1354	**1266**	**1005**
**984**	1266	1196	1567	**1624**	1008
1189	**1200**	1019	**1200**	**1414**	1428
1496	1354	1298	1193	1260	1029
1022	**1291**	1510	1005	1432	**1200**
**1008**	1567	1542	**1291**	1291	1354
1365	1005	1144	1624	1659	988
1638	**1316**	1659	1407	**1474**	**1624**
**1200**	1624	1323	988	1418	1193
1347	1088	1358	1344	**1005**	1390
**1684**	1344	1074	1428	**1538**	**1474**
1513	1210	1140	1088	**1200**	**1538**

The bold values indicated the corresponding bands occurred in both single-task and multi-task settings for BRR, MRR, and HRR, respectively.

## Data Availability

The original contributions presented in this study are included in the article/Supplementary Materials. Further inquiries can be directed to the corresponding authors.

## Supplementary Materials

The following supporting information can be downloaded at: https://www.mdpi.com/article/10.3390/foods14111977/s1, Figure S1: Box-pot diagram for eliminating outliers; Figure S2: The distribution of measured values and the corresponding predicted values of the samples in the test set of the single-task BPNN model for BRR (a), MRR (b) and HRR (c); Figure S3: The distribution of measured values and the corresponding predicted values of the samples in the test set of the multi-task BPNN model for BRR (a), MRR (b) and HRR (c); Figure S4: Comparison of the top 20 shap values of (a) BRR, (b) MRR and (c) HRR on single-task and multi-task and the graph of Jaccard values of the top 20 shap values; Table S1: Experimental design and N-fertilizer management.
