# Supplementary material for: Nondestructive Detection of Rice Milling Quality Using Hyperspectral Imaging with Machine and Deep Learning Regression
Source: Foods. 2025 Jun 3;14(11):1977. doi: 10.3390/foods14111977 (PMC12155452; doi:10.3390/foods14111977)
Supplement: Supplementary file 1 [file foods-14-01977-s001.zip › foods-3634959-supplementary.pdf]

Supplementary Materials

Table S1. Experimental design and N-fertilizer management.

Experimental site	Treatment	Basal fertilizer (kg N hm−2)		Topdressing (kg N hm−2)		
		Methods	N rate	I	II	III
Balidian Station	N0	-	0	0	0	0
	FN240	SB	72	72	48	48
	F1N240	SB	240	-	-	-
	F2N240	SB	168	72	-	-
	F3N240	SB	168	-	72	-
	F1N192	SB	192	-	-	-
	F2N192	SB	134.4	57.6	-	-
	F3N192	SB	134.4	-	57.6	-
Jianliang Farm	N0	-	0	0	0	0
	CFN240	SB	72	72	96	-
	F1N240	SB	240	-	-	-
	F1N192	SB	192	-	-	-
	F1N168	SB	168	-	-	-
	MF1N240	MSDF	240	-	-	-
	MF1N192	MSDF	192	-	-	-
	MF1N168	MSDF	168	-	-	-

Note: SB, surface broadcasting; MSDF, mechanical side-deep fertilization; F, urea; F1-F3, controlled-release N fertilizer as basal fertilizer; CF, NPK briquette with a ratio of 16:16:16 as basal fertilizer; topdressing fertilizer I, at tillering stage; II, at panicle initiation stage; III, at booting stage, respectively. All treatments received 84 kg K2O hm−2 and 186 kg P2O5 hm−2 as basal fertilizer. N basal fertilizer occurred at the same time as being used in rice transplanting. The topdressing fertilizer was urea and applied by broadcasting.

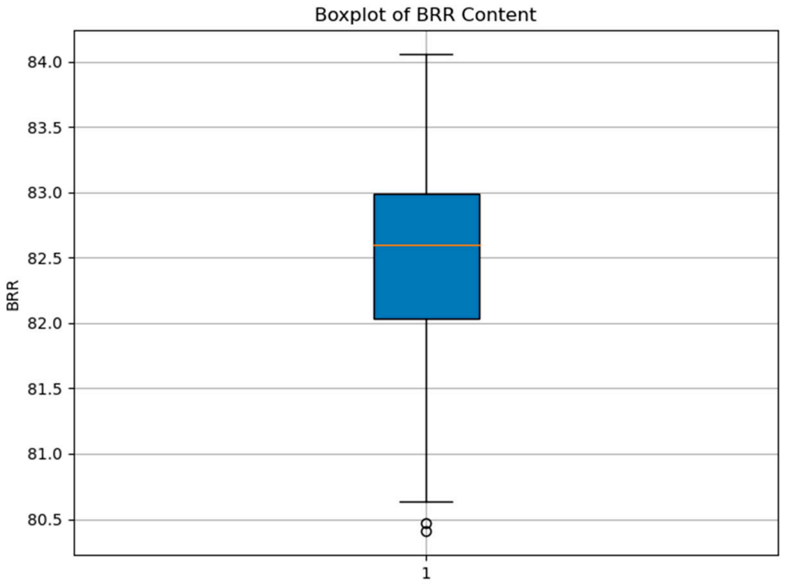

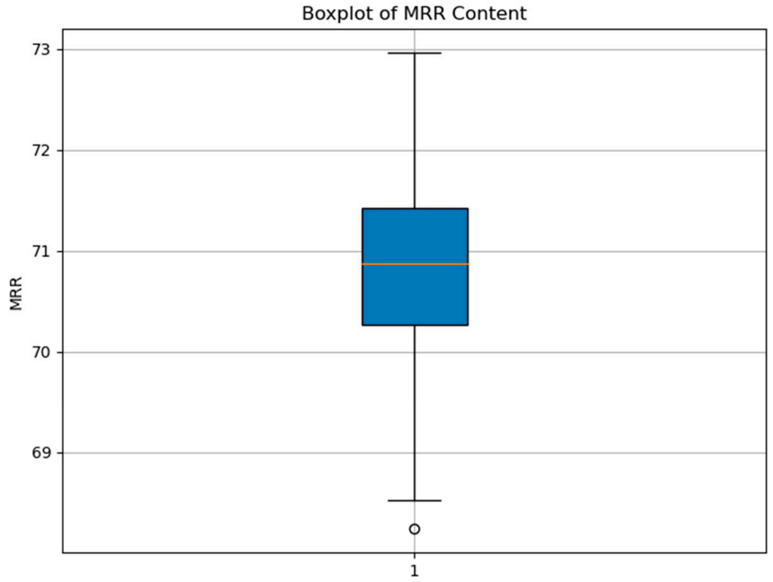

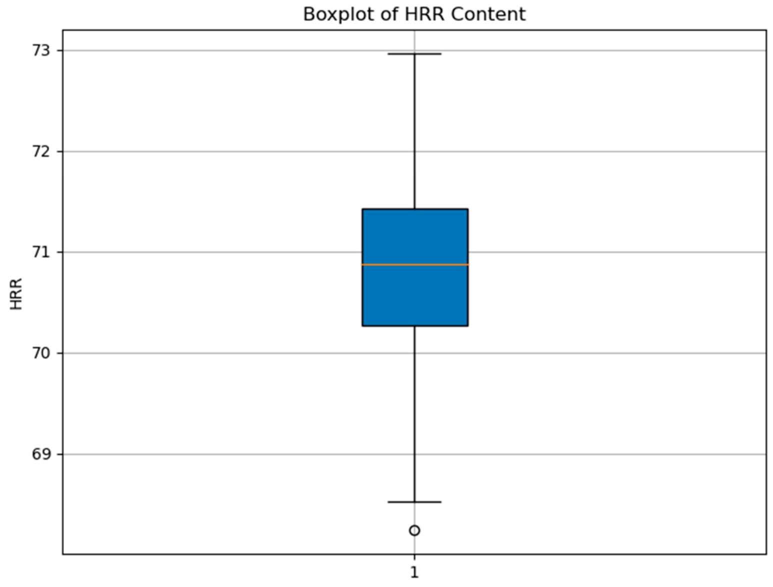

Figure S1 Boxplot diagram for eliminating outliers.

Figure S1. Boxplot diagram for eliminating outliers. For BRR, the samples of numbers 285 and 288 were identified as outlier samples, For MRR, the samples of number 288 were identified as outlier samples. For HRR the samples of number 288 were identified as outlier samples. Thus, the two samples of number 285 and number 288 were identified as outlier samples.

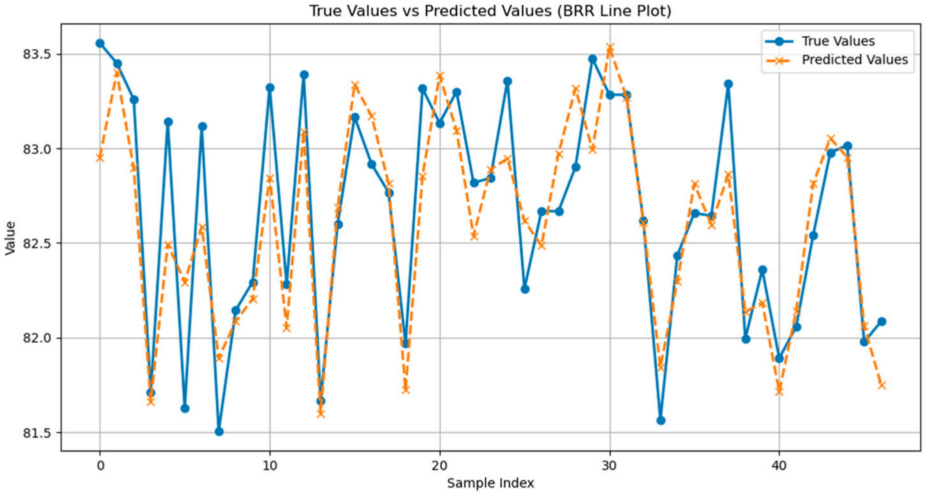
True Values vs Predicted Values (BRR Line Plot)
True Values
Predicted Values
Value
Sample Index
83.5
83.0
82.5
82.0
81.5
0
10
20
30
40

(a)

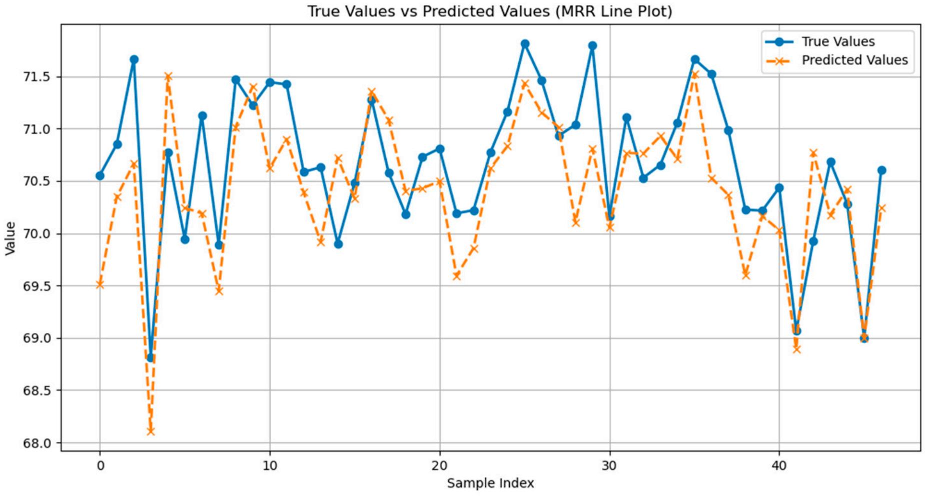
True Values vs Predicted Values (MRR Line Plot)
True Values
Predicted Values
Value
Sample Index
71.5
71.0
70.5
70.0
69.5
69.0
68.5
68.0
0
10
20
30
40

(b)

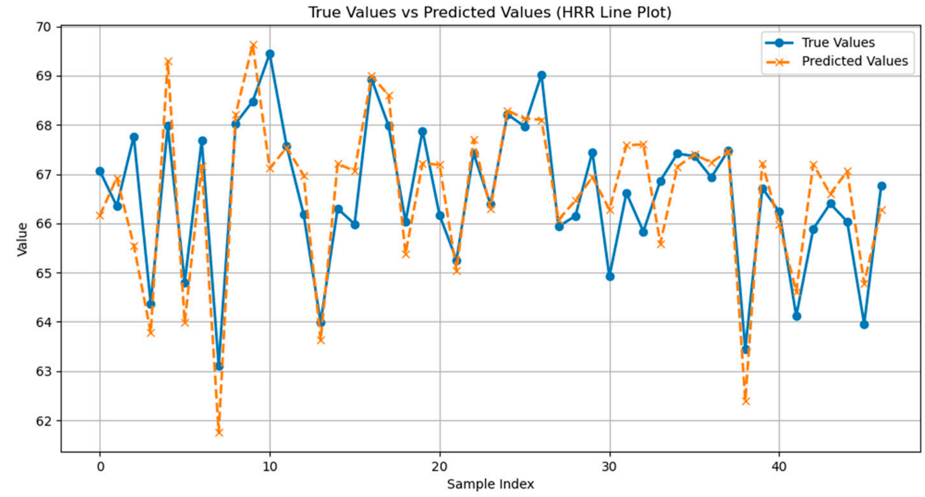
True Values vs Predicted Values (HRR Line Plot)
True Values
Predicted Values
Value
Sample Index
70
69
68
67
66
65
64
63
62
0
10
20
30
40

(c)

Figure S2. The distribution of measured values and the corresponding predicted values of the samples in the test set of the single-task BPNN model for BRR (a), MRR (b), and HRR (c).

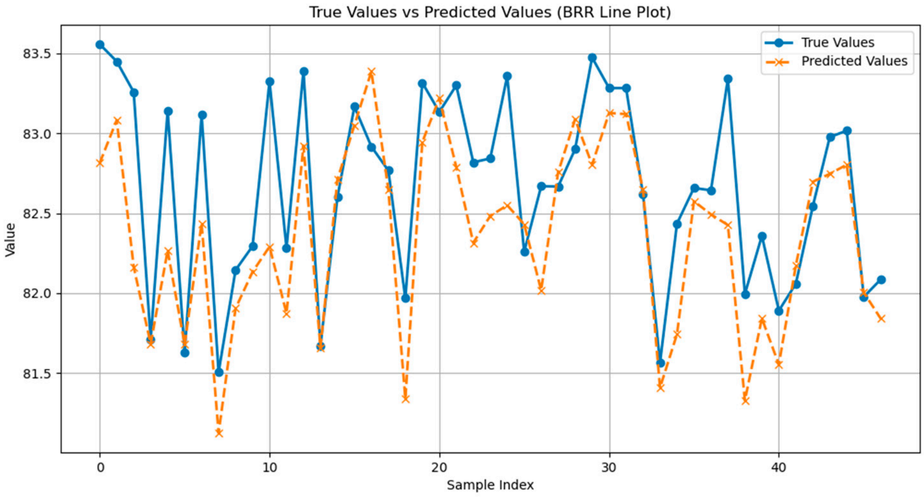

(a)

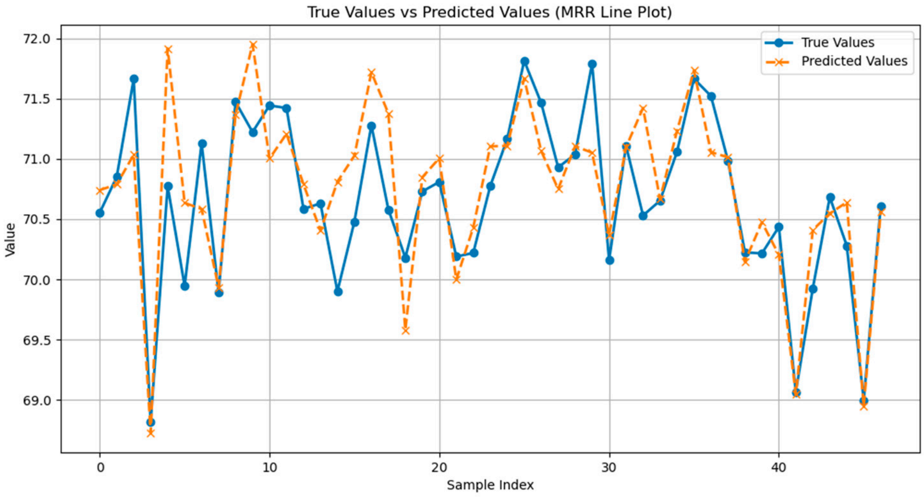

(b)

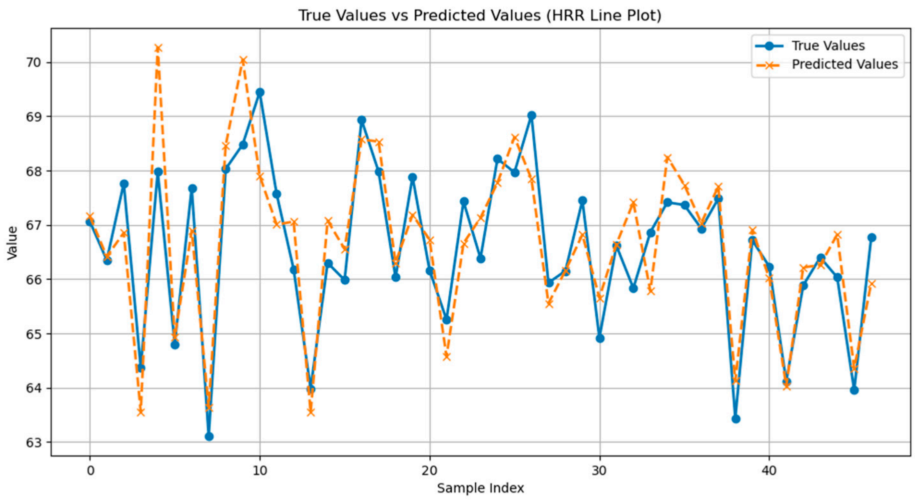

(c)

Figure S3. The distribution of measured values and the corresponding predicted values of the samples in the test set of the multi-task BPNN model for BRR (a), MRR (b), and HRR (c).

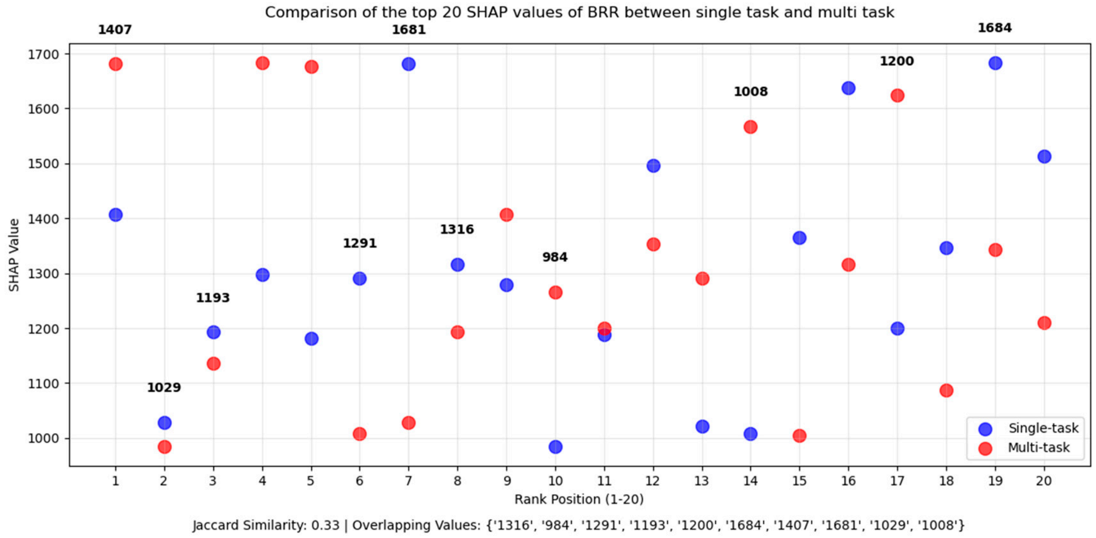

(a)

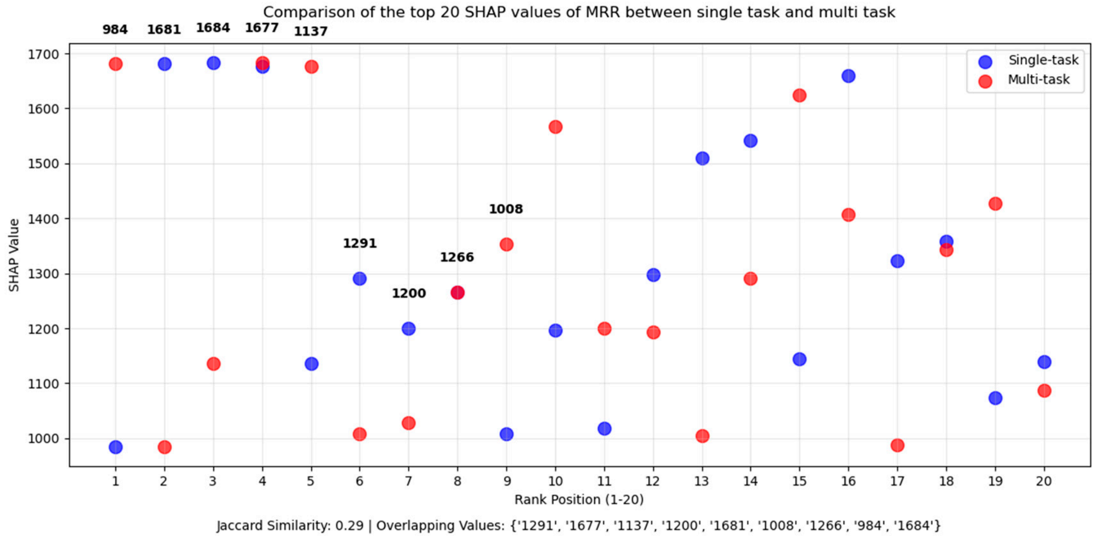

(b)

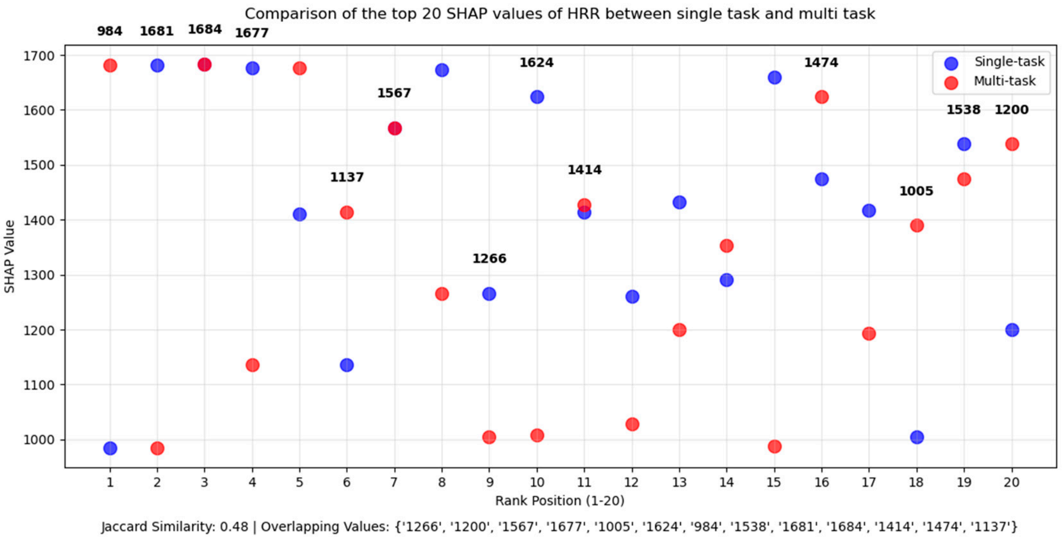

(c)

Figure S4. The Jaccard similarity analysis to compare the top 20 SHAP values of (a) BRR, (b) MRR, and (c) HRR in single-task and multi-task BPNN models. The blue point is the single-task SHAP value, and the red point is the multi-task SHAP value. The same SHAP value is displayed in bold font above the corresponding point. The Jaccard similarity coefficient and the specific overlap value are displayed at the bottom of the chart.
